# Supplementary material for: Cortico-subcortical β burst dynamics underlying movement cancellation in humans
Source: eLife. 2021 Dec 7;10:e70270. doi: 10.7554/eLife.70270 (PMC8691838; doi:10.7554/eLife.70270)
Supplement: Supplementary file 1. — For participants with Parkinson’s disease, scores shown are from the motor examination portion (i.e., total part III) of the Unified Parkinson’s disease rating scale (UPDRS). For essential tremor participants, scores are from the Fahn-Tolosa tremor scale. All Parkinson’s disease patients besides participant 2 were diagnosed with idiopathic Parkinson’s disease. Participant 2 had a diagnosis of both idiopathic Parkinson’s and essential tremor. [file elife-70270-supp1.docx]

| Participant number | Diagnosis | Implantation site | Handedness | Symptom laterality | Symptom severity score |
| --- | --- | --- | --- | --- | --- |
| 1 | Parkinson’s disease | STN | R | - | - |
| 2 | both | STN/VIM | L | left | 49 |
| 3 | Parkinson’s disease | STN | R | bilateral | 38 |
| 4 | Essential tremor | VIM | R | - | - |
| 5 | Parkinson’s disease | STN | R | left | 33 |
| 6 | Essential tremor | VIM | R | right | 47 |
| 7 | Essential tremor | VIM | R | right | 59 |
| 8 | Parkinson’s disease | STN | R | right | 37 |
| 9 | Essential tremor | VIM | R | left | 43 |
| 10 | Essential tremor | VIM | R | right | - |
| 11 | Parkinson’s disease | STN | R | right | 34 |
| 12 | Parkinson’s disease | STN | R | right | - |
| 13 | Essential tremor | VIM | R | bilateral | 78 |
| 14 | Essential tremor | VIM | R | right | 85 |
| 15 | Essential tremor | VIM | R | left | - |
| 16 | Parkinson’s disease | STN | R | right | 31 |
| 17 | Essential tremor | VIM | R | bilateral | 69 |
| 18 | Parkinson’s disease | STN | R | right | - |
| 19 | Parkinson’s disease | STN | R | left | 29 |
| 20 | Essential tremor | VIM | R | right | 66 |
| 21 | Essential tremor | VIM | R | bilateral | 57 |
